# Contributions to Acoustic Pathology

**Published:** 1848-07

**Authors:** James Mercer

**Affiliations:** Lecturer on Anatomy and Physiology, and on Diseases of the Ear, Edinburgh


*Contributions to Acoustic Pathology.* By JAMES MERCER, M. D., F. R. C. S. E., Lecturer on Anatomy and Physiology, and on Diseases of the Ear, Edinburgh.

PART III.—*On the Pathological Sequences of Acute Inflammation of the Fibro-mucous Structures of the Cavity of the Tympanum.*—TYMPANITIS—MYRINGITIS.

The inflammation that attacks the fibro-mucous structures of the cavity of the tympanum, may be either of a simple or isolated form, as when the membrana tympani is alone involved, or when it engages to a limited degree the membrane, and, more or less, the general investing membrane of the cavity; or it may be of an extremely complicated nature, more severe in its symptoms, and more fatal in its pathological sequences and results, in consequence of the numerous complications which this form of disease has always with all the deeper-seated parts of the organ.

The name of "*myringitis*" has recently been applied to this very complicated and dangerous form of disease of the middle ear, by Mr. R. A. Wilde, an experienced and scientific practical aurist of Dublin. In a paper published in the November number of 1847, of *The Dublin Quarterly Journal of Medicine*, this author has given the profession a most admirable and graphic description of the history, symptoms, and treatment of this formidable disease; and to this able production I would refer the readers of this paper, for the truthfulness of its description of the history, progress, symptoms, and treatment of it, both in the acute and chronic forms. It would be a mere repetition on my part, were I to attempt to add any thing to the above-mentioned production, as it is complete in itself; and, as I have stated at the commencement of this paper, I shall confine myself strictly to the pathological sequences resulting from this form of disease, myringitis. In this I do not wish to detract any thing from the credit due to Mr. Wilde; but, as a fellow-labourer in the same path in which he treads, I need only remind him, that "if we would treat of a science systematically and profitably, it is above all things necessary only to isolate it."—(*Feuchtersleben, Medical Psychology.*)

The pathological sequences which results from myringitis are very numerous, and by far the greater proportion of them are commonly fatal. I have endeavoured to arrange these, as simply and connectedly as the history, progress, and relative terminations of them enabled me to do; and of these I would enumerate the following list, which I have been able to glean from the records of science, or have seen in my own experience.

SECTION 1.—Caries of the Parietes of the Tympanum, producing Meningitis, without Destruction of the Petrous Portion of the Temporal Bone.

SECTION 2.—Caries of the Parietes of the Tympanum, producing Meningitis or Cerebritis, in consequence of destruction of the Osseous Septum between its Cavity and that of the Cranium.

SECTION 3.—Caries of the Parietes of the Tympanum, inducing Phlebitis of the Lateral Sinus and Internal Jugular Vein.

SECTION 4.—Caries of the Parietes of the Tympanum; Necrosis of the Petrous Portion of the Temporal Bone; Destruction of the Portio Dura, in the Aqueductus Fallopii, producing Paralysis of the Muscles of the Face.

SECTION 5.—Caries of the Parietes of the Tympanum; Necrosis of the Petrous Portion of the Temporal Bone; Destruction of the Gasserian Ganglion, producing Paralysis of Sensation in one half of the Face and Mouth.

SECTION 6.—Caries of the Parietes of the Tympanum; Necrosis of the Petrous Portion of the Temporal Bone; *Opening of the Internal Carotid Artery in its Canal of the Temporal Bone*, either alone, or in conjunction with the Lateral Sinus, or the destruction of the Gasserian Ganglion or the Facial Nerves.

I.—*Caries of the Parietes of the Tympanum, produciug Meningitis, without Destruction of the Petrous Portion of the Temporal Bone.*

The pathological connexion between the existence of diseases of the middle ear, and those of the membranes and substance of the brain, was for a long period unnoticed, and little attention was therefore paid to them; and it was not until within the last thirty years that special attention was directed to them, and their essential importance distinctly pointed out. The merit of the first improvement in this department of medicine is undoubtedly due to the late Dr. John Abercrombie, who, so early as 1821, directed the attention of the profession to the frequent occurrence of disease of the middle ear, as not only existing with, but generally preceding, inflammation of the dura mater, or the immediate investing membranes, or the substance of the brain. But even at this early period, Dr. Abercrombie did not press this important fact so forcibly on the profession as his subsequent experience enabled him to do. For in his case of meningitis of the cerebellum (case 15th, Ed. 2d, 1829) that proved fatal in 1821, the patient had laboured under all those symptoms which usually attend, and are characteristic of acute myringitis, and had also a discharge of purulent matter from the left ear very early in the disease. On inspection of the brain, it was found all healthy but the left lobe of the cerebellum. There, on its outer surface, was formed a uniform deposit of thick puriform matter, most abundant on the left side. The pia mater of the cerebellum was highly vascular; the dura mater was healthy; there was some purulent matter about the pituitary gland, and in the cavity of the middle ear, but *there was no appearance of disease of the bones connected with the ear, or of the dura mater covering them.*

This case we look upon as the most simple form—considering the pathological results—of the more extensive ravages which accompany, and are produced by acute myringitis. It is well known to all practical aurists who treat diseases of the ear on the principles of histological pathology, that in every case of acute myringitis considerable morbid changes always result to the parietes of the cavity of the middle ear, and no discharge of pus can take place from this cavity until the integrity of the membrana tympani becomes destroyed. It is unfortunate that no special account of the actual state of the parietes of the cavity of the middle ear has been recorded in the above quoted case, further than that "there was some purulent matter in the ear." Had the parietes of the cavity been more carefully examined; a greater extent of disease might have been detected. It is also well known, that if a person has once suffered from acute myringitis, and that this has been more or less successively relieved, that so long as any purulent discharge takes place from the external ear, the disease still exists in a chronic form; but if the patient becomes exposed to the influence of those agencies capable of reproducing the disease, it usually returns with all the force of an original attack.

I am inclined, therefore, to view this case as one of myringitis, and it is also further interesting, in showing that the disease of the membranes, or the substance of the brain, may result from diseases of the cavity of the middle ear, and without any destruction of the petrous portion of the temporal bone.

II.—*Caries of the Parietes of the Tympanum, producing Meningitis or Cerebritis, in consequence of destruction of the Osseous Septum between its Cavity and that of the Cranium.*

This is of more frequent occurrence than the former variety of cases, and is generally a very deceitful and insidious, but a most dangerous affection. It commences with all the symptoms of simple inflammation of the membrana tympani, or those of its more complicated form, myringitis; and many so affected consider it for a time as a trifling ear-ache. If a discharge of matter takes place from the ear, it is expected that the pain will be relieved; but on the contrary, it becomes more and more violent. The general course of such cases is, that the patient becomes drowsy and oppressed, delirium supervenes, shiverings, singultus and subsultus tendinum, and ultimately complete coma.

It is, also, not uncommonly found to occur in cases where acrid lotions have been employed to check suddenly the purulent discharge from the cavity of the tympanum, without any other counter-irritation having been adopted to prevent the occurrence of inflammation of the brain. In these cases the patient, after complaining for a day or two of having had deep-seated and very acute pain, especially during the night, in the ear, and along the face or side of the neck, suddenly becomes restless and forgetful—lies rolling his head from side to side, or tossing about his arms, and in a short time sinks into coma.

In both of these forms, the petrous portion of the temporal bone will be found to be more or less destroyed; and, as an illustration of the general course and termination of this form of disease, I shall quote the following case from Dr. Abercrombie:

"A gentleman, æt. twenty, on the 20th January, 1820, complained of violent toothach seated in a tooth in the right side of the upper jaw. On the 21st the pain extended into the ear, without any other symptom. On the 22d the pain continued in the ear, and extended towards the temple. He lay in bed part of the day, but got up afterwards. Leeches were applied, and he took some laxative medicine, which he vomited, and he had afterwards repeated vomiting. On the 23d the pain was more general over the head and across the forehead, with some vomitings, and at night shiverings. During the night be became incoherent and delirious; he was then seen by a surgeon, who found him very incoherent, but complaining of severe headach; the pulse 70, moderate in strength. Dr. Abercrombie saw him on the 24th; his pulse was then 60; his face rather pale; the headach continued, and was chiefly referred to the forehead; his look was vacant; he answered questions distinctly when he was roused, but talked incoherently when his attention was not kept up. He was now treated by general bleeding, which he bore well; cold applications, blistering, and purging. On the evening of the 24th there was considerable shivering. On the 25th, there was less complaint, but more incoherence, and a tendency to stupor; pulse 60—70. On the 26th, pulse 100 to 120. On the 27th and 28th little change; answered questions when roused, but, when not spoken to, lay in an oppressed state, or talking incoherently; pulse 96 to 120; some slight, but fetid, discharge from the ear. On the 29th, constant incoherent talking; pulse 96, of good strength; '*the right eye was suffused, the ball of it appeared turgid and enlarged, and the cornea was covered with a yellow slough.*' '*In the course of this day the mouth was, at times, observed to be drawn to the left side, especially when he was drinking.*' At night he began to sink, and died in the morning of the 30th.

"*Inspection of the Head.*—There was some effusion under the arachnoid on both hemispheres; much effusion into the ventricles, and extensive ramollissement of the septum lucidum, the fornix and the cerebral matter bordering on the lateral ventricles. There was extensive caries of the right temporal bone; behind the ear, on the thin part of the bone, it was very dark-coloured; and the petrous portion of the bone was dark-coloured, very soft; and when cut into discharged matter from its cancelli, and from the cavity of the middle ear. The dura mater corresponding to the temporal bone was much thickened. The part of it which lay anterior to the petrous portion was in a state of recent inflammation ; the part behind the petrous portion was much thickened and spongy ; and, between it and the bone, there was a deposit of thick purulent matter. From this place the disease had spread along the tentorium cerebelli, and nearly over the whole surface of the cerebellum, on almost every part of which there was a deposit of coagulable lymph, with thick, flocculent, purulent matter ; this was most abundant on the tentorium, and the right side and posterior parts of the cerebellum, and it was traced into the fourth ventricle. Under the cerebellum there was a considerable quantity of pus, and in its substance there was a small abscess, in the posterior part, between the lobes."

Such are the extensive ravages of this truly frightful disease ; and the two peculiar symptoms mentioned in the case,*" twisting of the mouth," no notice was taken of it in the above report of the *post-mortem* examination. This will be more fully alluded to in a subsequent section of this paper, in reference to those cases of destruction of the entire petrous portion of the temporal bone, causing thereby destruction of the facial nerve in the aqueductus Fallopii ; as also the " peculiar symptoms shown in the right eyeball," in connexion with the same destruction of bone, injuring the Gasserian ganglion, which lies upon its cranial surface.

### III.—*Caries of the Parietes of the Tympanum, inducing Phlebitis of the Lateral Sinus and Internal Jugular Vein.*

This is another, and by no means an unfrequent termination of complicated acute tympanitis, myringitis. In this class of cases, the osseous posterior septum of the mastoid cells gives way, and, immediately on its occurrence, the dura mater covering the point of diseased bone becomes diseased, presenting all the symptoms of meningitis. From the proximity of the sigmoid curve of the lateral sinus along the cranial surface of the mastoid cells, the lining membrane of the vein becomes speedily inflamed, and, extending rapidly along it to the heart, forms a fatal phlebitis of the internal jugular vein.

The following case, reported in the Reports of the Dublin Pathological Society, Vol. XIX., is one of the most interesting examples of this form of disease in the records of medicine.

" A boy, æt. sixteen years, entered the Hardwicke Hospital in Dublin, May 27, 1840, under the care of Mr. R. W. Smith. He had been exposed to the greatest hardships and laborious exertions from his earliest youth. He had been ill for seven days previously to his entrance into the Hospital ; he complained of shiverings, and a cold, creeping sensation, succeeded by intense pain in the right ear and right side of the face. He had a nausea and vomiting, with a loss of appetite ; he was constantly drowsy, and prevented from sleeping by a loud noise in his ear.

" After remaining under medical treatment for a short time, he left the hospital and resumed his work ; but was soon obliged to discontinue it from the debility and occasional syncope with which he was overpowered. When he was again admitted he could not walk steadily; he had no spasmodic or irregular action of the muscles, but he staggered from vertigo; he was thin and pale, and had a vacant stare, with large and equally dilated pupils; his answers to questions were slowly but rationally given; he complained of severe shooting pains through the back part of his head into the right ear, from which flowed a greenish, fetid matter; his tongue was white and moist; his pulse 132, sharp and small; and his skin was hot.

"He grew rapidly worse after his admission; he slept but little, started frequently from his sleep, moaning from the acute pain in his right ear; whenever he attempted to rise he supported his head with his hands, and was sensible of a noise in his head like the splashing of water; there was a sense of fluctuation and great tenderness over the mastoid process; a teaspoonful of fetid pus was given exit by incision, and the bone was found denuded of its periosteum; he had great epigastric tenderness and ardent thirst.

"Upon the 3d of June he had a jaundiced hue, and an attack of diarrhœa with tenesmus; he had also a distressing cough, and severe pain along the right side of the neck. Upon the 6th symptoms of arachnitis set in; violent, darting pain in the head; alternations of heats and chills; a rapid pulse; delirium; dilated and irregular pupils; vomiting; occasional singultus; he was restless; burning heat of scalp, and cold extremities; he soon became comatose, ceased to answer questions rationally, and died June 11.

"*Examination of the Head.*—The brain was firm; the left hemisphere pale; the right highly vascular in the interior, and the membrane covering it was minutely injected with blood, especially along its inferior surface. Three small purulent deposits, surrounded by a vascular circle, and apparently encysted, were found at the inferior surface of the right lobe of the cerebellum, where it corresponded to the lateral sinus. The dura mater was separated by pus and lymph of a green colour from the anterior surface of the petrous portion of the temporal bone; but there was no perforation of the membrane. Over that portion of bone which constitutes the superior wall of the tympanum, it was elevated into a small tumour by a collection of fetid matter, and presented a sloughy aspect. The portion of bone corresponding to this abscess, of a circular form, from about one fourth of an inch in diameter, was dead, and of a dull white colour. The process of separation from the living bone was far advanced, and at one point of its origin the separation was complete, and the aperture thus formed communicated with the cavity of the tympanum; the remainder of the petrous portion was remarkable for its vascularity; the membrana tympani had disappeared completely, and the membranous walls of the right lateral sinus, throughout the whole of the mastoid portion of its course, were much thickened, and the lining membrane of the vessel presented a sloughy appearance, being covered with lymph of a greenish hue, and smeared with unhealthy purulent matter. This condition extended along the internal jugular vein and superior vena cava, to within a short distance of the entrance of the latter vessel with the right auricle. The lining membrane of the vena cava was of a dead tawny colour."

In connection with this division of our subject, I will also quote the following case, as reported by Professor Syme in the March number of the Monthly Journal of Medical Science, 1841, p. 153, wherein the carotid artery was tied for hemorrhage from the external ear, and similar in its pathological cause to the above-mentioned case.

"In the spring of last year, Dr. James Wood asked Mr. Syme to see a young gentleman, eleven years of age, on account of an alarming hemorrhage from his ear. He was recovering from an attack of scarlatina, in consequence of which both ears had suppurated, when, upon the fifteenth day, a large quantity of blood was suddenly discharged from the right side. During the six succeeding days the bleeding returned three times, to the extent, by computation, of a pound on each occasion. It was deemed proper to place a ligature on the carotid artery, which was concluded to be the source of the hemorrhage. Bleeding recurred while the operation was being performed, and twice again to a small extent, not exceeding a few teaspoonfuls, in the course of the following evening and night.

"For several days afterwards, there was hardly any appearance of blood, and all the circumstances encouraged the entertainment of favorable hopes. Symptoms of cerebral excitement, however, then showed themselves, and terminated fatally on the eleventh day after the operation.

"On examination, it was found that the cartoid artery was *not* concerned in the disease, but that a small ulcerated aperture in the osseous septum, between the termination of the lateral sinus and the cavity of the ear, had permitted the blood to escape from this vessel.

"*Could this have been ascertained previously*, stuffing the ear would, of course, have suggested itself as the proper practice."

There is a foot-note in connection with the above reported case, in which Professor Syme refers to "a case of bleeding from the ear, in which recovery followed this operation" (tying the carotid artery.) I have consulted this case, and find that it has no pathological relation to the present section of cases. That case was, evidently, one of perforation of the internal carotid, before it had entered the canal in the petrous portion of the temporal bone, as the principal part of the blood that was discharged came from the back part of the pharynx. The *possible* symptom that was exhibited, *bleeding from the ear*, and leading to the supposition of perforation of the vessel, is distinctly shown from the results of the case to have been accidental. The blood that had been discharged into the pharynx would have been partly swallowed, and, during the primary effort at deglutition, the influence of the superior constrictor of the pharynx would carry the blood, also, into the pharyngeal opening of the Eustachian tube, and thence by it to the tympanum, where the membrana tympani, having been destroyed, it was discharged from the external ear.

It is unfortunate that, in the narration of the above case of Professor

Syme's, no notice was taken of the *physical properties* of the blood discharged. In cases belonging to the present section, where the symptoms are so doubtful and so deceitful, every trifling circumstance should be taken into consideration before a positive diagnosis is formed or acted on.

IV.—*Caries of the Parietes of the Tympanum; Necrosis of the Petrous Portion of the Temporal Bone; Destruction of the Portio Dura in the Aqueductus Fallopii, producing Paralysis of the Muscles of the Face.*

This form of complication with myringitis is of comparative rarity, and with the exception of two cases, accidentally mentioned by Dr. Abercrombie, one of which we referred to in the second section of the present paper, there is only another complete case on record, and reported by Dr. R. Graves, in the Dublin Journal, Vol. XX. I have met with one case also in my own experience; but it was complicated with loss of sensation (anæsthesia) of the face, and which I will notice in the next section.

The case of Dr. Graves is as follows:—

"A boy, about ten years of age, was admitted into the Meath Hospital labouring under general dropsy; he appeared of a scrofulous habit, and was much worn down by long-continued diarrhœa. Under appro priate treatment, his symptoms gradually, though slowly, disappeared, and he was restored to comparative health. We now observed that the right side of the face was paralysed, and on examination found that he had been subject to a discharge from the right ear for seven years previously. The paralysed cheek presented the phenomena usually oberved in Bell's Paralysis. He was attacked soon after with acute pain in the ear, and in the left side of the head. A fortnight after, convulsions set in; the pain moved from the side to the back of the head, then to the back of the neck, and ultimately extended the whole way down the spine, and about this period the diarrhœa diminished. A few days before his death he was attacked with spasms resembling those of tetanus, and the surface of the body became exquisitely tender to the touch. He never had any loss of motion, and to the last his intellect was perfect. From the period when the pain set in to that of his death, the convulsions returned six times.

"*Post-Mortem.*—The portio dura was dissected on the face, and found healthy; the nerve was also healthy from its origin at the base of the brain to the entrance into the meatus auditorious internus. Immediately above this opening the dura mater was of a greenish colour, detached from the bone as if by fluid, and perforated by a round hole, large enough to admit one small crow-quill. On dividing this part of the membrane, the space between it and the bone was occupied by a thick, greenish offensive pus, and the opening in the dura mater was observed to be opposite to the foramen in the petrous portion of the temporal bones, called the *aqueductus* vestibuli. This opening was much enlarged, and the bone of it was in a carious condition.

"The nerves at the base of the brain were bathed in this thick green pus, but the organ itself was every where healthy, and free from excess of vascularity. The arachnoid was thickened and opaque, and the pia mater not more injected than natural. The ventricles were not distended. The theca vertebralis was much distended by the same kind of matter, which flowed abundantly from any accidental puncture of the membrane. The matter was contained in the sac of the arachnoid, which membrane was quite healthy, and presented its usual glistening appearance ; no thickening or opacity in any part of its extent. The pia mater was also free from disease; all the attachments of the ligamentum dentatum remained unbroken. The spinal chord, on being slit up, presented no trace of disease. The roots of all the spinal nerves from the base of the brain were bathed in pus, the presence of which fluid on the surface of the brain and spinal chord, had no doubt irritated those organs, and occasioned the tetanic symptoms and the cutaneous tenderness. The portio dura was traced through the aqueductus Fallopii, about a quarter of an inch from its entrance; the nerve was completely cut through, and the petrous portion of the bone was extensively destroyed, and presented a mere shell. The membrana tympani, and all the internal ear were completely destroyed."

It may be further mentioned here, that the spot where the portio dura was cut through, corresponds exactly to the point where the great petrosal, or vidian nerve, joins the portio dura, and forms the intumescentia gangliformis.

I shall now proceed to consider the fifth section of cases, which, when they do occur in practice, are usually complicated with those of the fourth; viz. paralysis of sensation in one half of the face—*anæsthesia.*

V.—*Caries of the Parietes of the Tympanum ; Necrosis of the Petrous Portion of the Temporal Bone ; Destruction of the Gasserian Ganglion, producing Paralysis of Sensation in one half of the Face.*

When we find paralysis and distortion of the face, with loss of sensation of the parts, we have reason to suspect the disease within the head, even without the existence of any active morbid action in the cavity of the ear. These cases have been referred to by the late Dr. Abercrombie in his section on diseases of the nerves ; but he has not favoured us with any cases of anæsthesia of the face, produced by the previous existence of myringitis. His cases, however, are of great importance, and relate entirely to those of paralysis and anæthesia consequent on some morbid state of the membranes surrounding the exit of the nerves from the cranial cavity in the substance of the brain, at their points of origin or emanation, or in some part of their course for distribution.

The symptoms of such cases are from those of the special case in connexion with myringitis, which I shall relate in every respect similar to those described by Dr. Abercrombie. The case is as follows:

"A young girl, seven years of age, and of a strumuous habit of body, became affected with scarlatina anginosa in the summer of 1843.

She was the daughter of a travelling gipsy, and resided in a wretched hovel in one of the filthiest alleys in the south side of the town. I was called to see her in the course of one of my dispensary visits. It was on the sixth day of attack when I first saw her. The cutaneous eruption, which had evidently been very dark, was almost gone; there was great difficulty in breathing, a hoarse voice, sneezing, cough without expectoration, and an occasional slight hemorrhage from the nose. The surface of the tongue, and insides of the cheeks, were covered with numerous aphthæ; the tonsils were much swelled, but there was no evidence of decided gangrene, though there was considerable superficial ulceration on both sides. The child was delirious, and had been so for twenty hours, screaming wildly, and instinctively putting her hands to her right ear, the right side of her face, and neck. When she was coherent, she complained to her mother of a severe pain coming on in these parts, and, when I attempted to examine her ear, she instinctively indicated severe agony, and tried to thrust away my hand. A discharge of matter had taken place from the right ear four hours before I saw her; but the symptoms showed no relief. On examining the mastoid process it was larger than usual, discoloured, and had a slight feeling of softening and pitting. An incision made into it gave exit to a full teaspoonful of very fetid pus; but none of the small bones, or any gritty particles, could then be found in that discharged matter, or in that coming from the outer ear. A large warm linseed meal poultice was applied to the right ear and side of the face; two grains of calomel, and three grains of Dover's powder, were ordered to be given every four hours, and, in the intervals, a teaspoonful of weak wine and water.

"On the morning of the second day there had been a decided increase of all the cerebral symptoms; the wine and water had been swallowed with difficulty, and part of it ejected again. A small enema of Ol. Terebinth and gruel, that had been exhibited the previous night, had operated well in emptying the bowels. The discharge still continued, both from the outer ear and the incision in the mastoid process, and, on examining the concha, I found the malleus and incus bones, with the stapes attached to the latter, there amongst the discharge. Several gritty pieces of bone were also picked out from that of the mastoid process; and I fully concluded that complete destruction of the ear bulb had taken place, and that necrosis of the petrous portion of the bone would follow. No palsy of the muscles of the face as yet; but difficulty in swallowing. The eye-ball appeared larger than before, and had a dull look. A feather gently rubbed upon it still gave sensation, by a sluggish twinkling of the eyelids. Continued the medicines.

"At six, P. M. that day, I again called, and found the cerebral symptoms the same. There was more incoherence, and extreme restlessness; she tossed about her hands and legs, and, whilst I was present, she had a short convulsion. There was now distinct paralysis of the muscles of the face; greater difficulty in swallowing; the eyeball appeared still larger, and seemed to be starting from the orbit. It had become deeply congested, and was quite insensible to the irritation of the feather. The skin of the right side of the face might be pierced or pricked, but no sensation was evinced. The inside of the same cheek was in a similar state. I rubbed a little strong salt along the inside of the cheek, and along the right side of the tongue, but no evidence of any sapid body being there was shown; and a similar result followed the giving of a little powdered colocynth. On the left side of the face, however, there was distinctive evidence of sensibility remaining both to pricking, salt, and colocynth; and the eyeball there was also fully sensitive and apparently healthy. There was a slight fetid and bloody discharge from the right nostril. On examining the aperture of the mastoid, I found a spongy-looking mass of bone impacted in the incision there. This I carefully removed by a slight enlargement of the opening (the mastoid bone was very soft and easily cut,) and removed a great part of the mass of the petrous portion of the temporal bone. I bathed, then, the ear very gently with a sponge saturated with tepid water; gave her a little pure wine, and ordered a beef-tea enema. All the symptoms, as I left, were gradually increasing in severity.

"On washing carefully this necrosed portion of bone, I found it still to possess the conformation of the natural bone; its substance, however, was converted into a spongy mass, and the osseous labyrinth of the ear-bulb formed but a general part of the cancellated structure of it. Early on the third morning I found that, shortly after I had left, the convulsions came on with great frequency and violence; shiverings repeatedly; singultus, and ultimately coma, and death about four o'clock A. M. A dissection was granted.

"*Post-mortem appearances.*—To be careful in our examination, we succeeded in securing the entire head, stuffing up its place neatly, and leaving it apparently entire. On removing the calvarium and the dura mater corresponding to it, we found but a trifling sub-arachnoid effusion of opalescent lymph. No serum in the sac of the arachnoid there, but some congestion of the vessels of the pia mater on the upper surfaces of both hemispheres of the cerebrum. On slicing off these, there were a few bloody points here and there, similar to those found in cases of simple congestion of the veins of the cerebral substance. The lateral ventricles contained about two drachms of serum, and the septum lucidum and fornix were much softened. The choroid plexuses were much congested. On removing the entire nervous mass, we found the dura mater covering the upper surface of the petrous portion of the temporal bone very much diseased; it was elevated, soft, and spongy, of a dullish colour, and apparently on the point of becoming gangrenous. No distinct aperture was found in it, and it was raised up solely in consequence of the cavity from which the necrosed bone had been discharged, that cavity being completely filled with pus, and, floating on its surface, we found the Gasserian ganglion in a state of perfect destruction. The facial nerve was also found destroyed at its entrance into the aqueductus Fallopii, and was found so until the lower part of the stylo-mastoid canal. The whole of the osseous labyrinth had been destroyed and discharged; the osseous portion of the Eustachain tube that opens into the cavity of the tympanum was entire, but evidently diseased, and the internal carotid artery was not affected. Had the diseased action but continued for a few hours longer, the septum between this vessel and the tympanum would have been destroyed, and the vessel would have been opened. None of the tympanic muscles, vessels, or nerves, could be found; the osseous septum between the cavity and the sigmoid groove for the lateral sinus was entire, and no effects had been produced in the jugular vein.

"The inferior surface of the right middle lobe of the cerebrum, that lay upon the affected temporal bone, was highly inflamed, and much softened; there was a considerable effusion of lymph at the inner extremity of the right fissure of Sylvius, around the chiasm of the optic nerves, the tuber cinereum, the corpora albicantia, and the locus perforatus posterior, placed between the cura cerebri. The vascularity extended along the right crus cerebri to the mesocephalon, and thence, by the right crus cerebelli, to its right hemisphere. To all these parts the lymphy effusion was chiefly confined, and there was also some fluid in the cerebellar fossæ, the greater part of which had escaped by the removal of the head. The eyeball had not gone on to complete disorganization; but every part of its interior structure showed distinctive evidence that it was far advanced in a state gangrene. The vitreous body, and all within the iris, were converted into one confused mass.

"On dissecting the right nassal fossa and the pharynx, I found the Schneiderian membrane there in a state of extensive ulceration, not only in the general cavity, but also in all the facial cavities. The tonsils and side of the pharynx were also ulcerated; but the pharyngeal opening of the Eustachian tube, though also much ulcerated, was considerably entire. The left side was also much affected, but does not deserve a special description."

Such were the appearances seen in this interesting case; and I shall only add a few remarks in reference to its importance. It was remarked by Dr. Abercrombie (p. 447,) *loc. cit.*, "that a remarkable circumstance connected with the affections of the fifth nerve, is the tendency to inflammation and sloughing in parts which have lost their sensibility—particularly in the eye." Dr. Abercrombie relates a case that occurred to Dr. Alison, in which these results on the eyeball were very distinct; but the pathological cause did not belong to the present class of cases, as it was consequent on diseases between the Gasserian ganglion and the origin of the nerve at the mesocephalon.

VI.—*Caries of the Parietes of the Tympanum; Necrosis of the Petrous Portion of the Temporal Bone; Opening of the Internal Carotid Artery in its Canal of the Temporal Bone, either alone, or in conjunction with the Lateral Sinus, or the Destruction of the Gasserian Ganglion or the Facial Nerves.*

From the pathological sequences which I have shown as resulting from the ravages of complicated acute tympanitis, it will be easily understood that the above section of cases can easily form one of their number. The situation of the internal carotid in the canal of the petrous portion of the temporal bone, is not so secure in the nature of its position, or in the thickness of its osseous defences, as not to warn us that, some time or other, it will share alike in its destruction, as a sequence of myringitis, similar to what has so frequently occurred to the lateral sinus, and to the fifth and seventh pairs of nerves. There are several vulnerable points in the course of the artery in the canal of the bone, and the wonder is, that not one single case of its destruction has been put on record, so far as I can find; but that it is just as liable to destruction as any of the others are, is our decided conviction.

As I cannot present a single complete case to the profession in reference to this section, I must now conclude my remarks on this subject, by trusting that some more favoured observer will yet meet with such a case, and thus complete more fully the melancholy list of sequences that may follow acute tympanitis.—*Monthly Journal.*

